# Success in the Dental Profession

**Published:** 1862-05

**Authors:** C. C. Dills

**Affiliations:** Piqua, Ohio


### SUCCESS IN THE DENTAL PROFESSION.

BY C. C. DILLS, D. D. S.

### NO. I: CULTURE.

> "Can rules or tutors educate
> The semi-god whom we await?"

ALTHOUGH young, I have sought much for the secret of success in dentistry, that I might apply it to my own case, or aid the dental student in determining his qualifications, or the unsuccessful practitioner in his failings.

While I see much that is similar between success in ours and every other business, we yet have one somewhat peculiarly distinctive; but, what is it? At one time, I think, good sense; at another, professional skill; and at another, energy and *knack*—the great desideratum. Then, again, that success is purely constitutional, and may or may not embrace its usual elements, and may or not be attainable by all.

> "'Tis not in mortals to command success;
> But we'll do more, Semproneous—we'll deserve it;"

and, sooner or later, merit is apt to be rewarded.

To deserve success, is a duty; success itself is a gift. The duty is ours; the gift is ulterior. The empiric may disregard the duty, and yet succeed, as the vulgar regard success; but only the honest and faithful receive the benefit of a deserving success. The success of the former is insufficient, circumstantial, and capricious; the deserving of the latter is a perpetual source of gratification. "Applause waits on success," but much more fully on the deserving one.

A hurried anxiety to command success, beyond a point to keep us *doing*, endangers this deserving one. All great men have learned the worth of patient *industry*.

As a gymnast, looking to his full development for success, is ever ready to detect a weak muscle, and direct special attention thereto; so should we study the elements commanding success, and wherein we may find ourselves wanting direct special culture to the part.

This suggests the one grand element of success to be, culture, as embracing the full development of those faculties bearing upon our business.

Before noticing these faculties in detail, let me speak of culture comparatively.

And what does this "semi-god" not comprehend? By it we are to continually improve—detecting, modulating, or eradicating the animal and inferior—supplanting with the ever and anon appearing advance. By it we are to bring strength out of weakness; order out of confusion; aye, determine our own events for ourselves. And what does it not accomplish? Or rather, what success shall limit culture? Freedly plainly attributes all success to it; and Emerson says it is the "ambition" of the age. Windship is its last demonstration. To culture, do we owe the genuine success of the individual everywhere, in science, art and literature. And I trust, through assiduous culture alone, the dental profession shall achieve its success.

But passing from this general view of the subject, let me consider it more definitely, and in detail. And, not to dwell on the ordinary qualifications of general and dental education, without which it is to be hoped none will enter the profession. I propose, in a series of articles, to group together and present to the reader those elements of success in the dental profession which, though less generally regarded, will be found happily blended in the successful dentist.

These articles will be severally entitled:

1. Affability, Urbanity, Tone;
2. Sympathy, Tenderness, Patience;
3. Frankness, Honesty, Truth;
4. Genius, Health, Energy;
5. Self-Reliance, Confidence, Concentration.

To develop and bring into perfect harmony and tone these weak muscles of our professional structure will be peculiarly to secure the esteem and suffrage of those around us, and "a cheerful, intelligent face, the end of culture, and success enough."

Piqua, Ohio, May, 1862.

